# Portable model for vasectomy reversal training

**DOI:** 10.1590/S1677-5538.IBJU.2019.0092

**Published:** 2019-01-29

**Authors:** Luis Otávio Amaral Duarte Pinto, Charles Alberto Villacorta de Barros, Anderson Bentes de Lima, Deivid Ramos dos Santos, Herick Pampolha Huet de Bacelar

**Affiliations:** 1 Programa de Mestrado Profissional em Cirurgia e Pesquisa Experimental Universidade do Estado do Pará Belém PA Brasil Programa de Mestrado Profissional em Cirurgia e Pesquisa Experimental, Universidade do Estado do Pará - Uepa, Belém, PA, Brasil

**Keywords:** Vasovasostomy, Vas Deferens, Fertility

## Abstract

**Objectives:**

To validate an experimental non-animal model for training of vasectomy reversal.

**Materials and Methods:**

The model consisted of two artificial vas deferens, made with silicon tubes, covered by a white resin, measuring 10 cm (length) and internal and external diameters of 0.5 and 1.5 mm, respectively. The holder of the ducts is made by a small box developed with polylactic acid, using a 3D print. The objective of the invention is to simulate the surgical field of vasovasostomy, when the vas deferens are isolated from other cord structures. For validation, it was verified the acquisition of microsurgical skills during its use, in a capacitation course with 5 urology residents from a Hospital of the region. Along the training sessions, it was analyzed the time (speed) of microsurgical sutures, and quantification of the performance using a checklist. Collected data were analyzed using de BioEstat®5.4 software.

**Results:**

Medium time for the completion of microsurgical sutures improved considerably during the course, and reached a plateau after the third day of training (p=0.0365). In relation to the checklist, it was verified that during capacitation, there was significant improvement of the scores of each participant, that reached a plateau after the fourth day of training with the model (p=0.0035).

**Conclusion:**

The developed model was able to allow the students that attended the course to gain skills in microsurgery, being considered appropriate for training vasectomy reversal.

## INTRODUCTION

Vasectomy is a safe and efficient contraceptive method. Worldwide, it is estimated that nearly 60 million men had been submitted to this procedure (1). According to DATASUS database, only in November 2018, 3,127 surgeries were performed in public health services in Brazil (2).

Although widely accepted, men submitted to vasectomy may seek reversal of fertility due to death of children, divorces, new relationships, among other life circumstances (3). It is estimated that 6% of vasectomized men look for fertility reversal sometime in their lives (4).

Among possible options, vasectomy reversal (vavovasostomy) is considered the gold standard procedure, with patency and pregnancy rates of up to 89.4% and 73.0% respectively (5).

In order to perform vasovasostomy, urologists must train skills in microsurgery, mastering the use of optical microscope and very delicate surgical instruments, made with cutting-edge technology (6). Unfortunately, most Brazilian public services lack those equipment, and an expressive quantity of urologists finish their residences without learning microsurgical skills, and have to spend money and time in capacitation courses.

Some authors advocate the use of experimental models for training and gaining microsurgical skills. Grober et al. (7) and Shurey et al. (8) developed models of vasovasostomy training using laboratory mice, with good results of capacitation. However, pressure of society to lower the use of experimental animals along with restrictions for their use by only some selected research centers are stimulating the development of artificial models, that reliably simulate *in vivo* surgical procedures (9).

The first artificial vasovasostomy model was described by Li et al. in 1992 (10). It consisted of the use of microsurgical sutures in silicon tubes. In that time, the author used tubes with 1.5 mm of internal diameter, higher than the human vas deferens lumen, that varies from 0.4 to 0.7 mm, limiting its importance (11).

Therefore, there is a need to produce training models for vasovasostomy that are affordable, that spare the use of experimental models and that mimic efficiently human vas deferens. Such models could be introduced to training urologists to capacitate them in microsurgery, filling that formation gap.

The objective of the present study is to validate an experimental non-animal model, developed for the training of vasectomy reversal.

## MATERIALS AND METHODS

### Ethical aspects

The study was developed according to Helsinki statements and Nuremberg Code, respecting the rules of researches with human beings.

### Model development

The training model for vasectomy reversal was developed at the Experimental Surgical Laboratory of University of the State of Pará (LCE-UEPA). Artificial vas deferens ducts were made of translucid silicon tubes, measuring 10 cm width, with internal and external diameters of 0.5 and 1.5 mm respectively. They were covered externally with a white PVA resin film (vinil poliacetate), allowing the simulation of all vas deferens layers, such as lumen mucosa, and muscular and adventitia layers.

**Figure 1:**
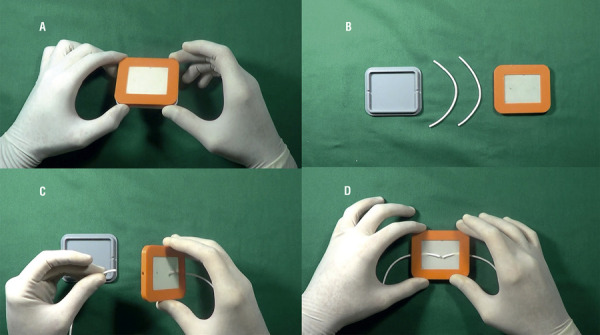
Training model for vasectomy reversal (A and B: model components; C and D: assembling of the components).

**Figure 2:**
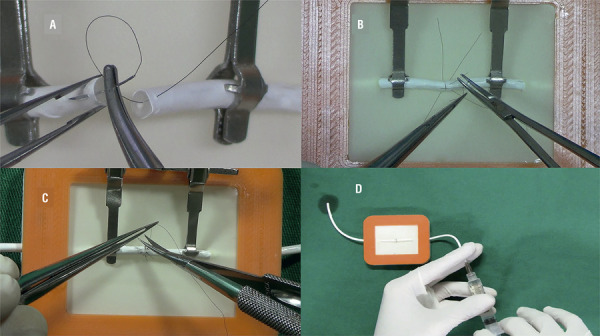
Vasovasostomy in a training model (A: stitches applied through all duct layers; B and C: microsurgical sutures; D: proof of a patent anastomosis).

The holder of the artificial ducts was made by a small box (with base and cover) developed with polilatic acid using a 3D printer Makerbot®. The base has a non-slip cover, assuring adhesion and firmness of the device during training sessions. The cover has a rectangular 4.5x3.5 cm opening, made with latex, containing two small orifices through which the ducts are exteriorized, with stability during realization of the sutures. Figure-1 shows the components of the experimental model and the steps for mounting. Figure-2 shows a vasovasostomy performed using the device.

### Validation of the model

In order to validate the model, it was analyzed the gain of skills in microsurgery during training. It was proposed a study with 5 urology residents from a reference public hospital of the region, and none had previous experience with microsurgery.

The participants were submitted to a capacitation workshop in microsurgery, using the previously described model. The course consisted of a first day of first impressions (DO), followed by 5 training sessions, with weekly intervals (D7, D14, D21, D28 and D35).

At DO, the residents watched a theorical 30 minutes video demonstrating basic aspects of microscope use, positioning and operatory technique, followed by practice with microsurgical sutures using training plates, with 1 hour duration.

The other sessions (D7 to D35) involved the performance of vasovasostomies using the experimental model. In the beginning and in the end of each session, the residents performed two microsurgical sutures in the training plate (each with a double semi-knot and two simple semi-knots), that were named pre-training, post-training and vasovasostomy.

Also, during the training sessions, the participants were also evaluated with a checklist (Appendix), that assigned a score according to the performance during surgery.

### Materials and recommended technique

For the microsurgical suture in the model it was used: microsurgical needle holder Castroviejo 10 cm width without rack; dissection clamp watchmaker straight, 10 cm width; curved Castroviejo scissors, 10 cm width, and microspike clamp. It was also used Nylon 8-0 suture, with two spatulated1/4 0.65 cm needles. Anastomosis was made using stereoscopic magnification by a conventional optical microscope D.F. Vasconcelos®.

Recommended vasovasostomy technique is characterized by 4 simple equidistant sutures, englobing all layers of the model, at 3, 6, 9 and 12 hours; interleaved with 4 sutures that spared the lumen, allowing complete coaptation of all circumference, according to the recommended technique described by Benlloch et al. (12).

### Statistical analysis

Analytic parameters were processed in the software Microsoft Excel® and Word® 2013 creating tables and graphics that posteriorly were submitted to statistical analysis using BioEstat® 5.4 software. Data were initially submitted to Shapiro Wilk normality test. For normal distributed data it was used ANOVA parametric test. For those with abnormal distribution it was used the Kruskal Wallis non parametric test. And finally, for comparison, it was used the paired t-Student. Statistical significance was set at p≤0.05.

## RESULTS

**Figure 3:**
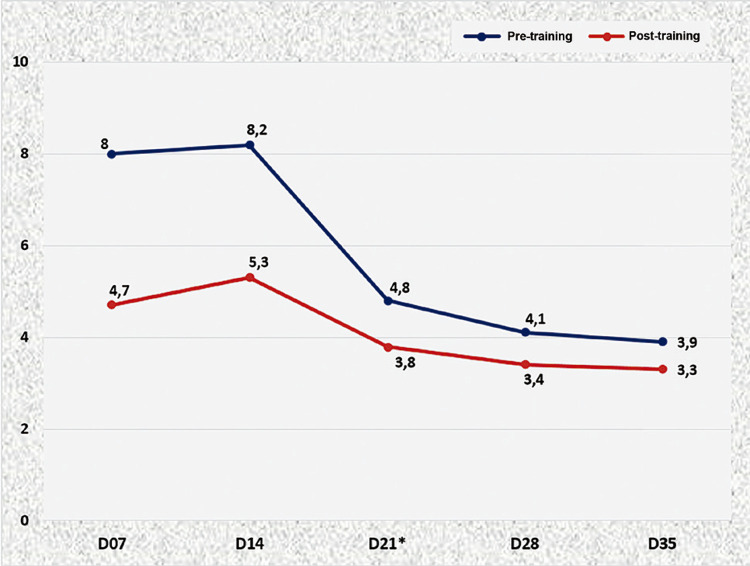
Medium time, in minutes, of sutures during pre and post-training throughout all sessions.

In Figure-3 shows a graphic comparison between time of suture media of all participants, between pre-training and post-training, in all training sessions. It is observed that the medium time for complete suture improved considerably during the 5 days of the course, as in the end of each session.

* Paired t-Student test (p=0.0365)

When D7 was analyzed singly (first capacitation session, using the model) it is observed an important improvement between the post-training time and respective pre-training time. This transitory gain in skills became more consistent along the other sessions, which is demonstrated by the graphic showing approximation of the curves pre- and post-training. After the third day of training (D21), the participants reached a plateau, consolidating the gain of skills in microsurgery (p=0.0365).

**Figure 4:**
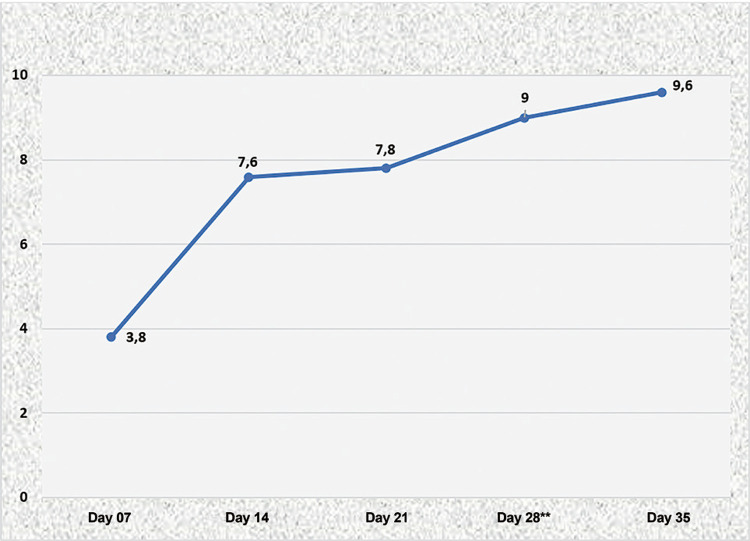
Medium cheklist score throughout training sessions.

In Figure-4 shows the progression of skills of residents in microsurgery, using the media results obtained with the checklist throughout capacitation. The analysis of the graphic shows an important increase of score right after session two (D14), that remains progressive until reach a plateau on the fourth session of training (D28), with a medium score of 9 points (p=0.0035).

** ANOVA test one criteria (p=0.0035)

## DISCUSSSION

The objective of this study was to validate a non-animal experimental model to develop skills in microsurgery, particularly vasectomy reversal. The model simulates the surgical field, when the ducts are isolated from other elements of the spermatic cord.

The model in the shape of a small box is easy to handle and storage, and can be reused several times; the used segment is severed and the stubs are approached again. It is estimated the model allows for 35 vasovasostomies.

The coat with a layer of PVA simulates the different layers of the vas deferens, allowing for different sutures (total or partial layers). Benlloch technique was chosen since it is considered the easiest available for urologists in initial training.

The course, with 5 training weekly sessions, is similar to most microsurgery courses with international relevance (13). Evidences show that acquired skills is higher when there is an interval between session, in comparison to consecutive day training (14).

Literature demonstrates that direct evaluation of training in experimental models (artificial or animal) are highly reliable for microsurgery training (15, 16). Temple et al. (17) and Grober et al. (18) state that this kind of evaluation may be performed using timely parameters, as well as the use of checklists or scales.

In the present study, model validation was checked with improvement of time spent for microsurgical sutures and the progressive increase of score of the checklist during capacitation. We believe that the use these two criteria allowed for more concise result interpretation than the analysis of only one parameter.

Results show a plateau of skill acquisition at the third session of training (D21), when only time was considered, and at the fourth session (D28), when checklist score was considered. These aspects reinforce the idea that evaluation with detailed and specific criteria (in a checklist or scale) represent more reliably the acquisition of skill throughout training, rather than only the analysis of time.

Analysis of time spent in this study was a complementary evaluation of acquired skills in microsurgery. We timed the suture time spent at pre and post-training instead that of vasovasostomy *per se*, based on the publication of Starkes et al. (19). According to these authors, time is secondary in the analysis of a good microsurgical anastomosis; other criteria are more important, such as correct handling of tissue, stitches applied equidistantly, good edge cooptation, among others. A “fast” vasectomy reversal is not always the “best”.

Therefore, time analysis only at the moment of microsurgical knots (at pre and post-training) allowed for an objective interpretation: better skills are observed with faster stitches.

The artificial model has some disadvantages: it simulates only the surgical field and does not allow for training the other steps of vasovasostomy, such as identification of buds, fibrosis section, and calibration of deferens lumen. Another unfavorable aspect is that silicon obviously does not present the same physical proprieties than human vas deferens; therefore, for a good coaptation and patency analysis, it was necessary to stabilize and align the stumps, with the aid of the microspike clamp, and the use of a double semi-knot at first, not necessary in real surgeries.

Our original idea when we proposed the current study was to broaden the teaching of microsurgery for urology residents, particularly those that work at public services with low budget. We hope that this study encourage the development of more realistic models for vasectomy reversal or other microsurgical procedures in Urology, such as varicocele correction, neophaloplasties, penile reimplantation, among others.

Literature reinforces the use of high definition video systems, that produces image magnification similar to surgical microscope, with the advantage of being much less expensive, needing only a camera and a TV set (20). We believe that this kind of technology may be used along with similar models , and that, in the future, urologists that intend to improve their microsurgical skills can do so at home, with reliable simulators, without the use of laboratory animals or the need to go to a hospital or facility that has a microscope.

## CONCLUSIONS

The developed experimental model was efficient to train vasectomy reversal, allowing for skills improvement in microsurgery.

## Appendix

**Table:** Checklist used for validation of experimental model.

Checklist - Experimental model for training vasectomy reversal												
**1.Did the resident remained in a comfortable position with forearms and wrists support?**												
[ ]D7		[ ]D14			[ ]D217			[ ]D28			[ ]D35	
**2.Did the resident cut the suture in half for better use?**												
[ ]D7		[ ]D14			[ ]D217			[ ]D28			[ ]D35	
**3.Did the microspike clamp was used for approaching the stubs?**												
[ ]D7		[ ]D14			[ ]D217			[ ]D28			[ ]D35	
**4.Did the resident handled correctly the microsurgical thread? (not using the needle)**												
[ ]D7		[ ]D14			[ ]D21			[ ]D28			[ ]D35	
**5.Did the resident make some sutures with backhand?**												
[ ]D7		[ ]D14			[ ]D21			[ ]D28			[ ]D35	
**6.Did the resident perform the 3 semi-knots?**												
[ ]D7		[ ]D14			[ ]D21			[ ]D28			[ ]D35	
**7.Did the resident perform 8 suture stitches? (4 total layer and 4 partial layer)**												
[ ]D7		[ ]D14			[ ]D21			[ ]D28			[ ]D35	
**8.Were the stitches equidistant?**												
[ ]D7		[ ]D14			[ ]D21			[ ]D28			[ ]D35	
**9.Were the suture stubs satisfactory? (not too short, not too long)**												
[ ]D7		[ ]D14			[ ]D21			[ ]D28			[ ]D35	
**10.Did the needle was kept at the sponge in order to avoid its loss?**												
[ ]D7		[ ]D14			[ ]D21			[ ]D28			[ ]D35	

**Onus/bonus:**												

**11.Did the resident lost the needle during training? (yes = -1 point)**				[ ]D7			[ ]D14		[ ]D21	[ ]D28		[ ]D35
**12.Did the needle was damaged during procedure? (yes = -1 point)**				[ ]D7			[ ]D14		[ ]D21	[ ]D28		[ ]D35
**13.Did the resident use only half the suture thread? (yes = +1 point)**				[ ]D7			[ ]D14		[ ]D21	[ ]D28		[ ]D35
**14.Did the vasovasostomy was patent? (yes= +1 point)**				[ ]D7			[ ]D14		[ ]D21	[ ]D28		[ ]D35

**Total score:**												

[ ]D7			[ ]D14			[ ]D21			[ ]D28		[ ]D35	

## References

[1] 1. Eisenberg ML, Lipshultz LI. Estimating the number of vasectomies performed annually in the United States: data from the National Survey of Family Growth. J Urol. 2010;184:2068-72. 10.1016/j.juro.2010.06.117 20850832

[2] 2. DATASUS - Tecnologia da Informação a Serviço do SUS: Procedimentos hospitalares do SUS – por local de internação [citado 22 de agosto de 2017]. available at. <http://tabnet.datasus.gov.br/cgi/tabcgi.exe?sih/cnv/qiuf.def>.

[3] 3. Li PS, Ramasamy R, Goldstein M. Male infertility microsurgical training. In: Sandlow JI, editor. Microsurgery for Fertility Specialists. New York: Springer; 2012.

[4] 4. Potts JM, Pasqualotto FF, Nelson D, Thomas AJ Jr, Agarwal A. Patient characteristics associated with vasectomy reversal. J Urol. 1999;161:1835-9. 10332448

[5] 5. Crain DS, Roberts JL, Amling CL. Practice patterns in vasectomy reversal surgery: results of a questionnaire study among practicing urologists. J Urol. 2004;171:311-5. 10.1097/01.ju.0000100801.40282.b0 14665903

[6] 6. Parekattil SJ, Gudeloglu A, Brahmbhatt J, Wharton J, Priola KB. Robotic assisted versus pure microsurgical vasectomy reversal: technique and prospective database control trial. J Reconstr Microsurg. 2012;28:435-44. 10.1055/s-0032-1315788 22744901

[7] 7. Grober ED, Hamstra SJ, Wanzel KR, Reznick RK, Matsumoto ED, Sidhu RS, et al. Laboratory based training in urological microsurgery with bench model simulators: a randomized controlled trial evaluating the durability of technical skill. J Urol. 2004;172:378-81. 10.1097/01.ju.0000123824.74075.9c 15201815

[8] 8. Shurey S, Akelina Y, Legagneux J, Malzone G, Jiga L, Ghanem AM. The rat model in microsurgery education: classical exercises and new horizons. Arch Plast Surg. 2014;41:201-8. 10.5999/aps.2014.41.3.201 PMC4037763 24883268

[9] 9. Weber D, Moser N, Rösslein R. A synthetic model for microsurgical training: a surgical contribution to reduce the number of animal experiments. Eur J Pediatr Surg. 1997;7:204-6. 10.1055/s-2008-1071093 9297513

[10] 10. Li PS, Schlegel PN, Goldstein M. Use of silicone medical grade tubing for microsurgical vasovasostomy training. Urology. 1992;39:556-7. 10.1016/0090-4295(92)90017-q 1615608

[11] 11. Middleton WD, Dahiya N, Naughton CK, Teefey SA, Siegel CA. High-resolution sonography of the normal extrapelvic vas deferens. J Ultrasound Med. 2009;28:839-46. 10.7863/jum.2009.28.7.839 19546325

[12] 12. Ramada Benlloch FJ, de la Torre Abril L, Tramoyeres Galvañ A, Cánovas Ivorra JA, Sánchez Ballester F, Ordoño Domínguez F, et al. [Our experience with simplified vasovasostomy. Review of our results during the last 5 years]. Arch Esp Urol. 2004;57:59-63. 15112872

[13] 13. Leung CC, Ghanem AM, Tos P, Ionac M, Froschauer S, Myers SR. Towards a global understanding and standardisation of education and training in microsurgery. Arch Plast Surg. 2013;40:304-11. 10.5999/aps.2013.40.4.304 PMC3723987 23898423

[14] 14. Moulton CA, Dubrowski A, Macrae H, Graham B, Grober E, Reznick R. Teaching surgical skills: what kind of practice makes perfect?: a randomized, controlled trial. Ann Surg. 2006;244:400-9. 10.1097/01.sla.0000234808.85789.6a PMC1856544 16926566

[15] 15. Kalu PU, Atkins J, Baker D, Green CJ, Butler PE. How do we assess microsurgical skill? Microsurgery. 2005;25:25-9. 10.1002/micr.20078 15645419

[16] 16. Ramachandran S, Ghanem AM, Myers SR. Assessment of microsurgerycompetency-where are we now? Microsurgery. 2013;33:406-15. 10.1002/micr.22111 23712917

[17] 17. Temple CL, Ross DC. A new, validated instrument to evaluate competency in microsurgery: the University of Western Ontario Microsurgical Skills Acquisition/Assessment instrument [outcomes article]. Plast Reconstr Surg. 2011;127:215-22. 10.1097/PRS.0b013e3181f95adb 21200214

[18] 18. Grober ED, Hamstra SJ, Wanzel KR, Reznick RK, Matsumoto ED, Sidhu RS, et al. Validation of novel and objective measures of microsurgical skill: Hand-motion analysis and stereoscopic visual acuity. Microsurgery. 2003;23:317-22. 10.1002/micr.10152 12942521

[19] 19. Starkes JL, Payk I, Hodges NJ. Developing a standardized test for the assessment of suturing skill in novice microsurgeons. Microsurgery. 1998;18:19-22. 10.1002/(sici)1098-2752(1998)18:1<19::aid-micr5>3.0.co;2-p 9635789

[20] 20. Sergio R, de Barros M, Brito MV, Leal RA, Teixeira RK, Sabbá MF, et al. A Low-Cost High-Definition Video System for Microsurgical Hindlimb Replantation in Rats. J Reconstr Microsurg. 2017;33:158-62. 10.1055/s-0036-1593767 27919114

